# Role of vitamin C and SVCT2 in neurogenesis

**DOI:** 10.3389/fnins.2023.1155758

**Published:** 2023-06-22

**Authors:** Katterine Salazar, Nery Jara, Eder Ramírez, Isabelle de Lima, Javiera Smith-Ghigliotto, Valentina Muñoz, Luciano Ferrada, Francisco Nualart

**Affiliations:** ^1^Laboratory of Neurobiology and Stem Cells, NeuroCellT, Department of Cellular Biology, Faculty of Biological Sciences, University of Concepcion, Concepcion, Chile; ^2^Center for Advanced Microscopy CMA BIO, University of Concepcion, Concepcion, Chile; ^3^Department of Pharmacology, University of Concepcion, Concepcion, Chile

**Keywords:** vitamin C, ascorbic acid, SVCT2, radial glia cells, neurogenesis, neuronal differentiation, pluripotency, epigenetic reprogramming

## Abstract

Different studies have established the fundamental role of vitamin C in proliferation, differentiation, and neurogenesis in embryonic and adult brains, as well as in *in vitro* cell models. To fulfill these functions, the cells of the nervous system regulate the expression and sorting of sodium-dependent vitamin C transporter 2 (SVCT2), as well as the recycling of vitamin C between ascorbic acid (AA) and dehydroascorbic acid (DHA) via a bystander effect. SVCT2 is a transporter preferentially expressed in neurons and in neural precursor cells. In developmental stages, it is concentrated in the apical region of the radial glia, and in adult life, it is expressed preferentially in motor neurons of the cerebral cortex, starting on postnatal day 1. In neurogenic niches, SVCT2 is preferentially expressed in precursors with intermediate proliferation, where a scorbutic condition reduces neuronal differentiation. Vitamin C is a potent epigenetic regulator in stem cells; thus, it can induce the demethylation of DNA and histone H3K27m3 in the promoter region of genes involved in neurogenesis and differentiation, an effect mediated by Tet1 and Jmjd3 demethylases, respectively. In parallel, it has been shown that vitamin C induces the expression of stem cell-specific microRNA, including the Dlk1–Dio3 imprinting region and miR-143, which promotes stem cell self-renewal and suppresses *de novo* expression of the methyltransferase gene Dnmt3a. The epigenetic action of vitamin C has also been evaluated during gene reprogramming of human fibroblasts to induced pluripotent cells, where it has been shown that vitamin C substantially improves the efficiency and quality of reprogrammed cells. Thus, for a proper effect of vitamin C on neurogenesis and differentiation, its function as an enzymatic cofactor, modulator of gene expression and antioxidant is essential, as is proper recycling from DHA to AA by various supporting cells in the CNS.

## 1. Introduction

Vitamin C, or ascorbic acid (AA), is a water-soluble hexose; at physiological pH, vitamin C ionizes predominantly in the form of a monovalent anion, called ascorbate, whose enodiol structure allows the donation of electrons that, after losing two electrons, forms the final oxidized product, dehydroascorbic acid (DHA) (Rice, 2000; Ngo et al., 2019). Thus, vitamin C is an important antioxidant against reactive oxygen and nitrogen species that are naturally produced during cell metabolism (Harrison and May, 2009). DHA can be reduced and recycled by glutathione or glutathione-dependent dehydroascorbate reductases (Maellaro et al., 1994). Vitamin C also acts as a cofactor in different enzymatic reactions involved in the synthesis of catecholamines, carnitine, cholesterol, amino acids (tyrosine metabolism) and some peptide hormones (Harrison and May, 2009). One of the most recognized functions of vitamin C is to facilitate the hydroxylation of proline and lysine residues to allow proper intracellular folding of pro-collagen to be secreted from the cell as mature collagen, strengthening blood vessels, the skin, muscles and bones (Przybylo and Langner, 2020; Ramirez et al., 2022; Thaler et al., 2022).

A new role has been assigned to vitamin C through specific DNA demethylation and consequent alteration of the expression of a set of genes in human embryonic stem cells (Chung et al., 2010; Cimmino et al., 2018), enhancing the reprogramming of human and mouse somatic cells into induced pluripotent stem cells (iPSCs) (Esteban et al., 2010; Chen et al., 2013; Gao et al., 2014; Cheng et al., 2015). These genomic effects of vitamin C are attributed to its role as a cofactor for the enzymatic activity of many Fe^2+^- and α-ketoglutarate-dependent dioxygenases (Monfort and Wutz, 2013; Cimmino et al., 2018), including key epigenetic regulators such as histone demethylases Jhdm1a/1b (Wang et al., 2011) and DNA demethylase ten-eleven translocation (TET1) (Blaschke et al., 2013; Chen et al., 2013; Cheng et al., 2015; Han et al., 2021; Crake et al., 2022).

## 2. Vitamin C and its transporters

Two isoforms of vitamin C and sodium transporters, SVCT1 and SVCT2, have been identified (Daruwala et al., 1999; Tsukaguchi et al., 1999). Isoforms expressed in human tissues share 65% amino acid sequence identity but display different functions due to their differential expression (Daruwala et al., 1999; Tsukaguchi et al., 1999). In this sense, SVCT2 has a higher affinity for its substrate but lower transport capacity than SVCT1 (Savini et al., 2008). At the tissue level, SVCT1 is mainly confined to the surface of epithelial cells (Tsukaguchi et al., 1999; Castro et al., 2008; May, 2011) and participates in the transport of vitamin C in the intestine, kidney, liver, lung, and other tissues (Luo et al., 2008; Nualart et al., 2013; Subramanian et al., 2017). SVCT2 is preferentially expressed in specific tissues, such as the brain, adrenal and pituitary glands, lymphoid tissue, muscle, and bone (Tsukaguchi et al., 1999); however, basal levels of SVCT2 are also found in other tissues. Thus, SVCT1 regulates gastrointestinal absorption and renal reabsorption (Forman et al., 2020), maintaining a plasma vitamin C concentration of 50 μM. Vitamin C can also be incorporated into cells using glucose transporters of the GLUT type; however, these transporters incorporate the oxidized form of vitamin C, dehydroascorbic acid (DHA) (Nualart et al., 2003).

**FIGURE 1:**
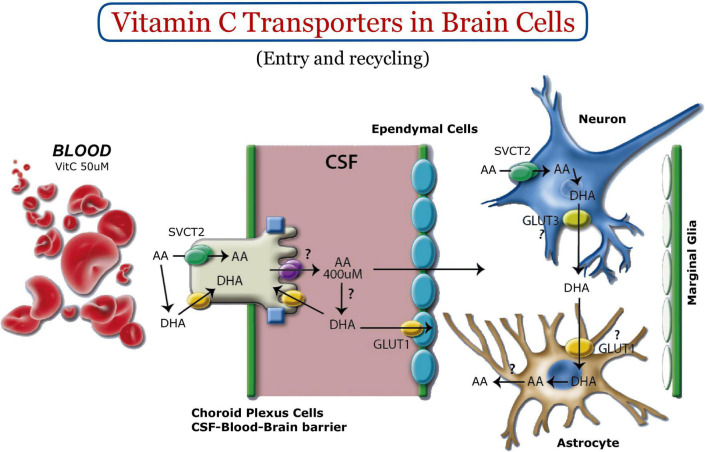
Vitamin C homeostasis in the nervous system. Vitamin C enters the CSF through the choroid plexus. SVCT2 and GLUT1 are expressed basolaterally (green and yellow, respectively). GLUT12 (purple, apical) could be the apical polarized transporter for the transport of vitamin C within the CSF (Miyata et al., 2022). Once vitamin C is concentrated in brain tissue, it is incorporated into neurons using SVCT2. Intracellular oxidation generates DHA, a molecule that can leave neurons through GLUT3. DHA is incorporated into astrocytes to be reduced to AA and released back into the extracellular space. Most astrocytes do not express SVCT2; however, marginal glia have been shown to express it (Nualart et al., 2012).

In the adult CNS, SVCT2 is detected in neurons of the cerebral cortex, hippocampus, hypothalamus, and cerebellar Purkinje cells (Garcia Mde et al., 2005; Mun et al., 2006; Nualart et al., 2012; Oyarce et al., 2018; Figure 1). We have demonstrated high levels of SVCT2 expression in pyramidal neurons of the inner region of the cerebral cortex, mainly between the postnatal stages P1 and P5 (Salazar et al., 2014). Regarding its expression in glia, SVCT2 is detected in microglia (Garcia Mde et al., 2005; Mun et al., 2006; Portugal et al., 2017), ependymal cells and tanycytes (Garcia Mde et al., 2005; Mun et al., 2006), and Schwann cells (Gess et al., 2010). With the exception of astrocytes located in the marginal region of the cerebral cortex (marginal glia) that express SVCT2 (Nualart et al., 2012; Figure 1), SVCT2 is not present in most parenchymal astrocytes (Berger and Hediger, 2000). Instead, SVCT2 expression is induced when brain tissue is under stress (Berger et al., 2003; Salazar et al., 2018) or in astrocytes isolated for culture *in vitro* (Berger and Hediger, 2000). SVCT2 has been shown to be basolaterally polarized in choroid plexus cells (Ulloa et al., 2013, 2019), confirming the participation of the choroid plexus-CSF barrier in the entry of vitamin C into the brain (Angelow et al., 2003). This generates a vitamin C concentration of 500 μM in CSF, and additionally, a concentration of 200–400 μM in the extracellular fluid (Figure 1). SVCT2 has not been detected at the blood-brain barrier; thus, the entry of vitamin C into the brain by this route has been ruled out (Berger et al., 2003; Spector and Johanson, 2006). Regarding the neurogenic areas, SVCT2 is expressed in the fetal rat brain, mainly located in the ventricular and subventricular areas, where the precursor cells (radial glia) form the neurogenic niche (Nualart et al., 2012; Silva-Alvarez et al., 2017). Due to the location of the neurogenic niches in the brain, the cells that form these structures will have practically direct access to vitamin C, which is highly concentrated in the brain. Considering that SVCT2 is a high affinity transporter (Km 20 μM), this transporter will always be in saturated conditions for the incorporation of vitamin C. Thus, the intracellular levels of vitamin C will be regulated intracellularly by the presence or not of SVCT2 in the stem cell membrane.

Sodium-dependent vitamin C transporter 2 (SVCT2)-knockout mice die shortly after birth, with undetectable brain levels of vitamin C and diffuse hemorrhages in the cerebral cortex (Sotiriou et al., 2002; Harrison et al., 2010) and areas lower brainstem (Harrison et al., 2010). Interestingly, the embryos do not show bleeding in other tissues and do not have signs of generalized scurvy (Sotiriou et al., 2002), but they do have increased oxidative stress in various organs and reduced type IV collagen in the basement membranes of the brain (Harrison et al., 2010).

## 3. Vitamin C and SVCT2 in stem cells of the developing nervous system

### 3.1. SVCT2 expression and localization in radial glial cells

During the development of the nervous system, stem cells called radial glial cells (RGCs) are generated from the neuroepithelium before neurogenesis begins (Alvarez-Buylla et al., 2001; Noctor et al., 2001; Doetsch, 2003; Kriegstein and Gotz, 2003; Gotz and Barde, 2005; Malatesta et al., 2008). The “Radial Glia” is an elongated cell whose body covers the entire thickness of the wall of the neural tube, with an apical process that contains the nucleus and that is oriented toward the central cavity and a long basal process that contacts the external surface (Alvarez-Buylla et al., 2001; Noctor et al., 2001; Doetsch, 2003; Kriegstein and Gotz, 2003; Gotz and Barde, 2005; Malatesta et al., 2008). In this mitotically active cell, interkinetic migration of the nucleus occurs during the S phase of the cell cycle, and its self-renewal can be symmetrical, generating two daughter cells with the characteristics of the initial cell (neural stem cell), or asymmetric, generating a new radial glia and a cell that can be differentiated (progenitor cell of a cell subtype) (Alvarez-Buylla et al., 2001; Noctor et al., 2001; Doetsch, 2003; Kriegstein and Gotz, 2003; Gotz and Barde, 2005; Malatesta et al., 2008). Thus, it has been defined that in the development of the human nervous system, the radial glia generate neuroblasts (progenitor cells of neurons during neurogenesis) during the fourth to the twentieth week of embryonic development; the genesis of glial cells (a process called gliogenesis), such as astrocytes, oligodendrocytes, and ependymal cells, begins around week 20 and continues through the second year of life (Kratzing et al., 1985; Stiles and Jernigan, 2010).

**FIGURE 2:**
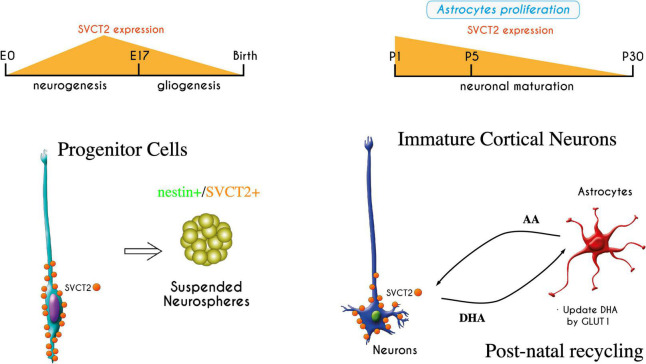
Expression of SVCT2 during embryonic brain development and in early postnatal stages. SVCT2 has been detected in radial glia, with apical polarization (Silva-Alvarez et al., 2017). Its expression varies during development, increasing in radial glia at the peak of neurogenesis. SVCT2 is also expressed *in vitro* when radial glia form neurospheres with nestin + cells. If neurospheres are attached to the substrate, the cells have processes that are strongly stimulated by vitamin C (Espinoza et al., 2020). In postnatal stages, the expression of SVCT2 increases again in the cerebral cortex; later, the transporter is detected primarily in motor neurons. In this period, the beginning of the recycling of vitamin C between neurons and astrocytes is necessary once the astrocytes are differentiated. If vitamin C recycling is disrupted, blocking GLUT1 function in astrocytes, neurons lose their processes (Salazar et al., 2021). The recycling of vitamin C is essential for neuronal development and arborization.

Recently, it has been shown that vitamin C has different effects on the stem cells of the nervous system, regulating their pluripotency and neuronal differentiation, which also depends on the functional expression of SVCT2 (Han et al., 2021). In this context, we report the early expression of SVCT2 in RGCs during the neurogenic period (E12-E17); this protein is detected in the ventricular pole that contacts the cerebrospinal fluid (CSF) (Silva-Alvarez et al., 2017). SVCT2 apical polarization is also induced when the protein is overexpressed using the *in utero* electroporation technique in the brains of E14 embryonic mice. It has also been observed in human brain tissue at 9 weeks of gestation (Silva-Alvarez et al., 2017). Interestingly, in the fetal brain of rodents, vitamin C levels double in the last gestational period (E15-E20), the period in which embryonic neurogenesis occurs (Kratzing et al., 1985; Figure 2).

### 3.2. Vitamin C and SVCT2 expression in isolated neural precursors

Treatment of J1ES cells, a cell model to study RGCs *in vitro* (Liour and Yu, 2003; Liour et al., 2006), with 200 μM AA for 12 h induces a radialized phenotype, promoting bipolar morphology and the expression of RGC markers, such as GFAP and RC2. This phenotype was even more potent when, along with AA treatment, the cells were cocultured in the presence of meningeal cells (Silva-Alvarez et al., 2017). This evidence demonstrates that vitamin C is necessary to maintain the state of RGCs and their eventual neurogenic potential.

Treatments with vitamin C support the hypothesis that vitamin C has a fundamental role in neurogenesis and gliogenesis. Additionally, treatment of CNS progenitor cells with Sonic Hedgehog (SHH) and fibroblast growth factor 8 (FGF8), in conjunction with AA, has been shown to significantly increase the number of dopaminergic and serotonergic neurons (Lee et al., 2003). Similarly, vitamin C increases dopaminergic differentiation of mesencephalic precursors (E12), which are maintained for long periods *in vitro* (Yan et al., 2001). Vitamin C works by upregulating a number of developmental genes to maintain the dopaminergic phenotype, such as Foxa2 and Lmx1a (early development); Nurr1 (intermediate development); and fully differentiated (TH +) DA neurons through removal of DNA methylation and the repressive histone code (H3K9m3, H3K27m3) (Wulansari et al., 2017). The treatment of embryonic cortical precursors supplemented with physiological concentrations of cerebral AA (200 μM) also increases the presence of astrocytes and neurons, where the latter show an increase in the frequency and amplitude of miniature excitatory postsynaptic currents (mEPSCs), suggesting that vitamin C could promote the acquisition of synaptic functions (Lee et al., 2003). In some of these reports, it has been determined that the cellular effects of vitamin C were accompanied by the continued expression of SVCT2 during the differentiation and proliferation periods of these neural precursors (Yan et al., 2001; Lee et al., 2003). In contrast, the absence of SVCT2 expression reduces the differentiation and synaptic maturation in cultured hippocampal neurons from animals deficient in SVCT2, which present stunted growth of neurites, less clustering of a-amino-5-hydroxy-3-methyl-4-isoxazole propionic acid (AMPA) receptor subunit GluR1 and reduced spontaneous neuronal activity (decreased frequency and amplitude of miniature excitatory postsynaptic currents) (Qiu et al., 2007).

Our group demonstrated the differentiating effect of increasing vitamin C uptake in neural precursor cells through the overexpression of SVCT2 (Salazar et al., 2016, 2021). N2a neuroblastoma cells treated with lentiviral vectors that overexpress SVCT2 develop an increase in the number of filopodia and MAP2-positive processes. This morphological change is not observable when lentivirus-treated cells are coincubated with an anti-SVCT2 antibody (to stimulate endocytosis of the transporter), with the inhibitor quercetin, or when the glucose transporter GLUT1 is overexpressed, which incorporates the oxidized form of vitamin C, DHA (Salazar et al., 2016). Similarly, the differentiating effect is not reproduced by overexpressing SVCT1 (a low affinity transporter and high transport capacity, which is not expressed in the brain), even when cells are supplemented with AA in the culture medium (Salazar et al., 2016). The gain of function of SVCT2 in cortical precursors isolated from embryonic brains (E14) induces an increase in arborization and increases the expression of synaptic proteins such as Piccolo and PSD95 and the presence of dendritic spines with thin and mushroom shapes, as observed by SR-SIM and 3D reconstruction rendering analysis (Salazar et al., 2021). This correlates with the expression of SVCT2 *in situ* in the mouse brain during the early postnatal period, where a strong induction of its expression is observed, which could be necessary to maintain the intracellular functions of AA on synaptic arborization and maturation in the pyramidal neurons of the internal region (layers VI-IV) of the postnatal cerebral cortex (PN1-PN5) (Salazar et al., 2014).

## 4. Function of vitamin C in adult neural precursors

### 4.1. Anterior lateral ventricle neurogénesis (subventricular zone)

Active neurogenesis occurs within the anterior wall of the lateral ventricle in the adult mammalian brain (Doetsch et al., 1999; Cebrian-Silla et al., 2021). The formation of new neurons, which are βIII-tubulin-positive, occurs in restricted compartments termed neurogenic niches (Doetsch et al., 1999; Obernier and Alvarez-Buylla, 2019). The neuroblasts formed in this region migrate tangentially in chains throughout the rostral migratory stream (RMS), where the presence of neurogenic progenitors and astrocytes has also been described. The neuroblasts present in the RMS reach the olfactory bulb, where they differentiate into interneurons (Lledo et al., 2008; Obernier and Alvarez-Buylla, 2019). B-type cells or astrocytes (GFAP- and nestin-positive) are preferentially located in the subventricular zone (SVZ) and are precursor cells. C-type cells are intermediate transient neuronal cells (nIPC) that proliferate rapidly and differentiate into neuroblasts or type-A cells (Eisch and Mandyam, 2007; Kriegstein and Alvarez-Buylla, 2009; Nualart et al., 2012; Obernier and Alvarez-Buylla, 2019). E-type cells, which are cube-shaped and multiciliated, are ependymocytes. B-type cells are found in the subependymal layer, projecting cilium to the ventricular lumen, similar to what has been described in the radial glia (Spassky et al., 2005); they also have a close relationship with blood vessels (Mirzadeh et al., 2008).

Using confocal immunofluorescence microscopy and *in situ* hybridization analysis, SVCT2 has also been detected in the subventricular zone (SVZ) and rostral migratory stream (RMS) of adult rats, specifically in proliferating BrdU + C-type cells and in neurospheres isolated from adult SVZ (Pastor et al., 2013). In isolated neurospheres, SVCT2 maintains its expression, where it was concluded that vitamin C induced neural differentiation increased βIII-tubulin and SVCT2 expression (Pastor et al., 2013). Furthermore, it has recently been shown that AA has a powerful differentiating effect, even with greater activity than retinoic acid in isolated neurospheres (Espinoza et al., 2020). However, AA is oxidized to DHA in long incubation periods, generating a loss in the formation of neurites. Surprisingly, neurite growth is maintained over time following co-incubation of neurospheres with cells that efficiently capture DHA. In this sense, astrocytes have high capacity to recycle DHA (see next section) and stimulate the maintenance of neurites. Thus, it was demonstrated that vitamin C recycling *in vitro* regulates the morphology of immature neurons during the differentiation and maturation processes (Espinoza et al., 2020).

Recently was demonstrated impaired neurogenesis in the SVZ of the brain of young vitamin C-deficient guinea pigs. The number of neuroblasts in the SVZ and subventricular zone lateral (SVL) extension of the lateral ventricle (equivalent to the RMS in guinea pigs) decreases progressively in guinea pigs exposed to a diet deficient in vitamin C for 14 and 21 days. The reduction reaches approximately 50% after 3 weeks of deficiency. By analyzing BrdU labeling, it was shown that the reduction in the number of neuroblasts corresponds to a decrease in cell proliferation in the SVZ (Jara et al., 2022). It is worth mentioning that there is no decrease in the survival of the new neurons, since the optical and electron microscopy images do not show an increase in the number of apoptotic cells (Jara et al., 2022). However, in the same work, it was shown that vitamin C deficiency produces morphological alterations in cells of the neurogenic niche of young guinea pigs, especially in the ependymal cells of the EVL (Jara et al., 2022). Taken together, this evidence demonstrated the importance of vitamin C in the proper development of new neurons in different neurogenic niches of the prenatal and adult brain.

### 4.2. Hippocampal neurogenesis

The subgranular zone (SGZ) of the hippocampal dentate gyrus (DG) is a neurogenic niche of the adult brain that contains neural stem cells (NSCs). In the neurogenesis in the SGZ, radial type 1 cells give rise to type 2 cells (transit-amplifying progenitors, nestin + and Sox-2 +), that differentiate into type 3 neuroblasts (doublecortin +) that become immature neurons and then mature into granule neurons that migrate into the granule cell layer (Kempermann et al., 2004; Varela-Nallar and Inestrosa, 2013).

Although there are no studies on the distribution of SVCT2 in SGZ of the hippocampal DG, it has been observed that long-term treatment with high levels of D-galactose reduces hippocampal neurogenesis and cognitive functions; however, cotreatment with vitamin C effectively prevents the reduction in hippocampal neurogenesis by enhancing cell turnover, neuronal differentiation, and neuronal maturation (Nam et al., 2019). Guinea pigs on a diet deficient in vitamin C show a significant difference in the Morris water maze (MWM) platform retention test, indicating a reduced ability of these animals to apply their spatial memory to the platform surface. The results of the MWM test are related to a significant decrease of approximately 30% in the number of neurons in the subdivisions of the hippocampus: the dentate gyrus, the CA1 area, and the CA2-3 areas, without distinguishing whether this decrease is due to increased death or decreased proliferation (Tveden-Nyborg et al., 2009).

Prenatal deficiency of vitamin C in guinea pigs produces a permanent deterioration in the postnatal development of the hippocampus (Tveden-Nyborg et al., 2012), producing a significant reduction in its volume, which is not reversed by reintroducing vitamin C postnatally. In BrdU labeling, there are no differences in proliferation or survival rates in the hippocampus; however, a significant decrease in the migration of newborn cells into the granular layer of the hippocampal dentate gyrus in prenatally deficient animals is observed (Tveden-Nyborg et al., 2012). In a subsequent study, it was shown that prenatal vitamin C deficiency in guinea pigs did not affect the volume of the hippocampus or the intensity of labeling of βIII tubulin (a marker of immature neurons) in prenatal guinea pigs, suggesting that the deterioration in the development of the hippocampus appears later, in the postnatal period (Hansen et al., 2016). Prenatal vitamin C deficiency also does not appear to affect synaptic plasticity or CA1 neuronal morphology in young guinea pigs (Hansen et al., 2018).

### 4.3. Cerebellar neurogenesis and other niches

The cerebellar structure is a suitable model for studying neural differentiation because its cell types and migration pathways during embryonic and postnatal development have been well characterized. The fully mature, adult cerebellar cortex is a complex structure in which the dendrites of Purkinje neurons, Bergman glial fibers, and other small neurons located in the molecular layer establish different connections, however, during the first two postnatal weeks granular precursor cells (produced for radial glial cells) are located at the outermost edge of the molecular layer of the cerebellum (Pibiri et al., 2016).

The presence of SVCT2 has been primarily described in the adult cerebellum (Meredith et al., 2011), specifically in Purkinje neurons. Previously, SVCT2 expression in the radial glial cells of the cerebellar anlage during embryonic development was also identified (Caprile et al., 2009). During postnatal day 1, SVCT2 was detected in the outermost region of the cerebellar cortex, corresponding to the external granular layer (EGL), a highly proliferative germinal zone (Oyarce et al., 2018). The intermediate filament protein vimentin was used to detect the Bergmann fibers that run throughout the EGL. At post-natal 5, SVCT2 was heterogeneously distributed in the EGL. At post-natal 15, SVCT2 was absent from the EGL but was detected in a diffuse pattern in the molecular layer, co-localizing with βIII-tubulin and exhibiting no association with vimentin expression. The strongest reaction for the transporter was observed in the soma of Purkinje neurons and in their projections into the molecular layer, whereas minimal expression was detected in the inner GL (Oyarce et al., 2018).

The C17. 2 cells are mouse-derived multipotent neural stem cells isolated from cerebellum, which were immortalized by avian myelocytomatosis viral-related oncogene (v-myc) transfection (Snyder et al., 1992). Functional analyses of vitamin C uptake in C17.2 neural precursor cells (SVCT2 +) show the existence of a saturable uptake mechanism for AA, which is sodium-dependent (Km 40 μM) (Tsukaguchi et al., 1999; Castro et al., 2001; Salazar et al., 2014; Silva-Alvarez et al., 2017; Oyarce et al., 2018). When C17.2 neural precursor cells are used to generate neurospheres, the presence of RGC-type elongated cells increases, which colocalize nestin and SVCT2 (Oyarce et al., 2018). Supplementation with 400 μM AA or SVCT2 overexpression for a period of 9 days induces a drastic decrease in nestin expression, increasing the neuronal marker βIII-tubulin (Oyarce et al., 2018), which accounts for the neuronal differentiation of these cells in the presence of chronic AA treatment.

Very little is known about the effect of vitamin C and SVCT2 expression in other neurogenic niches. At the level of the third basal ventricle, tanycytes are positive for SVCT2 and actively incorporate vitamin C (Garcia Mde et al., 2005; Nualart et al., 2014); however, an association between vitamin C/SVCT2 and neurogenic cells (alpha-tanycytes or median eminence stem cells) has never been studied.

A cell that is generally present in the different neurogenic niches is the microglia. This cell has been shown to be important for neurogenic differentiation, for example in the hippocampus (Diaz-Aparicio et al., 2020). In parallel, it has been shown that microglia express SVCT2, however there are no studies that analyze the relationship between vitamin C, microglia and neurogenesis.

## 5. Recycling of vitamin C and its effect on neural differentiation

During neuronal development (embryonic period), AA uptake by SVCT2 is restricted to radial glia and is not found in immature neuroblasts (Silva-Alvarez et al., 2017). In contrast, between days 1–5 postnatally, a strong induction of SVCT2 is detected in the Golgi apparatus in pyramidal neurons of layers IV-VI that are the first to be born (days E11-E13) (Salazar et al., 2014). Thus, the expression of SVCT2 and the active incorporation of AA in neuroblasts begin to reach the cortical layers and arborize to establish synaptic circuits (Salazar et al., 2014, 2018, 2021). During this period, vitamin C recycling is established to maintain AA levels and reduce the parenchymal accumulation of DHA and prevent its harmful effects (Nualart et al., 2014). During the early postnatal period (P1-P20) (Figure 2), the vitamin C recycling mechanism is established between recently differentiated astrocytes and maturating neurons. This has been studied in cultures of cortical neurons that overexpress SVCT2 or in cultures of neurospheres treated with AA (overexpressing SVCT2). These cells were incubated with 100–200 μM AA, which maintains their arborization and SVCT2 distribution throughout all their neurites, when cocultivated with U87 cells, HL60 cells or cortical astrocytes, which are capable of recycling DHA (expression of GLUT1) produced in neurons (Espinoza et al., 2020; Salazar et al., 2021). In contrast, neurite growth is negatively affected in the absence of these recycler cells or when these cells are treated *in vitro* with the inhibitor WZB117 (blocker of DHA incorporation through GLUT1). Similarly, *in vivo* injection of WZB117 into the cerebral cortex induces a greater number of neurons with low arborization characterized by a smaller number of neurites and shorter length (Salazar et al., 2021). The negative effects of DHA accumulation on the growth of neurites in neurospheres have been linked to the redox imbalance that favors an increase in reactive oxygen species (ROS) production, inducing an irreversible process of protein oxidation, including the carbonylation of cytoskeletal proteins such as actin and tubulin, thus inhibiting the maintenance of neurites (Espinoza et al., 2020).

Previous studies have shown that DHA accumulation in neuronal cells induces rapid glutathione consumption, inhibits glycolysis, and activates the pentose phosphate pathway (PPP) (Cisternas et al., 2014). Similar results were demonstrated in colon cancer cells treated with DHA, one of the targets being the inhibition of GAPDH due to the increase in ROS and a decrease in glycolysis (Yun et al., 2015). Furthermore, more recently, it has been observed that the accumulation of DHA in N2a and HN33.11 neuronal cells, generated under conditions of cellular stress due to intracellular oxidation of AA, induces non-apoptotic neuronal death, called necroptosis (Ferrada et al., 2021). Normal concentrations of AA regulate the expression of fundamental proteins in necroptosis, such as receptor-interacting serine/threonine-protein kinase 1 (RIPK1) and mixed lineage kinase domain-like pseudokinase (MLKL). The activation of necroptosis by DHA in neurons results in bubble formation, loss of membrane integrity, and ultimately, cellular rupture (Ferrada et al., 2020). These data suggest that necroptosis is a target for cell death induced by vitamin C.

## 6. Molecular mechanisms that enhance the pluripotency and differentiation effects of vitamin C and SVCT2

There are an increasing number of reports regarding the intracellular mechanisms of AA and SVCT2, which promote pluripotency or differentiation and synaptic maturation; we highlight the following:

– **Gene expression modulator:** First, in the 2000s, it was described that the differentiating effect of vitamin C is not related to its antioxidant capacity; these effects were not reproduced by other antioxidants such as glutathione or vitamin E (Lee et al., 2003). Subsequent studies demonstrated that vitamin C directly induced changes in gene expression (Shin et al., 2004; Yu et al., 2004). Thus, it was shown that vitamin C increases the expression of genes associated with dopaminergic differentiation by inducing the demethylation of DNA and histone 3 (H3K27m3) in the promoter region of genes involved in neurogenesis, differentiation and neurotransmission, an effect mediated by Tet1 and Jmjd3 demethylases, respectively (He et al., 2015). The mechanism by which vitamin C maintains the pluripotency of mouse embryonic stem cells (mESCs) has been investigated in J1 cells, observing an increase in Nanog expression (Gao et al., 2013) dependent on JAK2/STAT2 phosphorylation and the consequent activation of this pathway (Wu et al., 2014). Similarly, AA or DHA block the loss of Nanog when cells are stimulated to differentiate with retinoic acid, which indicates that vitamin C is a potent molecule in the maintenance of ESC pluripotency and that its effect does not depend on its antioxidant activity (Wu et al., 2014). Additionally, vitamin C increases the acetylation of lysine 5 at histone 4 (acH4K5) and the expression levels of the pluripotency maintenance genes Oct4, Sox2 and Klf4 in blastocysts of embryos generated by somatic cell nuclear transfer (SCNT), demonstrating that vitamin C promotes an *in vitro* and *in vivo* increase in the development of pig embryos with SCNT (Huang et al., 2011). Similarly, it has been shown that vitamin C induces the expression of ESC-specific microRNA (Gao et al., 2014), including the Dlk1–Dio3 imprinting region and miR-143, which promotes ESC self-renewal and suppresses expression of the *de novo* methyltransferase gene, Dnmt3a.
– **Enzymatic cofactor.** One of the main functions of vitamin C is its role as a cofactor of enzymes such as α-ketoglutarate and iron-dependent dioxygenases (Fe^2+^/α-KGDDs), where it partially maintains the reduced state of the Fe^2+^ ion (Monfort and Wutz, 2013; Kuiper and Vissers, 2014; Young et al., 2015). Among these enzymes are the prolyl hydroxylases that participate in the synthesis of collagen (Mata et al., 1981; Rappu et al., 2019). We have recently reported the importance of vitamin C in collagen synthesis in the biology of glioblastomas, which incorporate high doses of oxidized vitamin C (DHA), which is directly related to an increase in the perivascular invasion of tumor cells and their metastatic and aggressive capacities *in vivo* (Ramirez et al., 2022). Other dioxygenases whose function depends on AA are the epigenetic regulators of histone and DNA methylation (Table 1). As members of the dioxygenase family, they are cytosine demethylases in DNA, RNA, and histones, such as the ten-eleven translocation enzyme (TET) dioxygenase family, DNA and RNA demethylases of the AlkB homolog (ALKBH) family and Jumonji C domain-containing histone demethylases (JHDMs) (Cimmino et al., 2018; Han et al., 2021). Specifically, biochemical studies suggest that, for example, for TET2, vitamin C (AA), but not other antioxidants, binds directly to the C-terminal catalytic domain and acts as a specific electron donor to restore iron to the Fe^2+^ state in the catalytic cycle of TET, thus increasing the oxidation rate of 5mC up to 8-fold in a dose- and time-dependent manner (Yin et al., 2013; Table 1).

**TABLE 1:** Vitamin C-associated enzymes involved in epigenetic reprogramming and pluripotency.

Family	Enzyme	Epigenetic modification	Substrate	References
TET	TET1	DNA demethylation	5mC, 5hmC, and 5fC	Blaschke et al., 2013; Chen et al., 2013; Ditroia et al., 2019
	TET2	DNA demethylation	5mC, 5hmC, and 5fC	Blaschke et al., 2013; Yin et al., 2013
	TET3	DNA demethylation	5mC, 5hmC, and 5fC	Ito et al., 2010
JMJC	JHMD1a/b	Histone demethylation	H3K36me2/3	Wang et al., 2011; Comes et al., 2013
	KDM3a/3b	Histone demethylation	H3K9me2	Ebata et al., 2017
	JHMD3	Histone demethylation	H3K27me3	Mansour et al., 2012; Huang et al., 2020; Ding et al., 2021; Lu et al., 2022
	ND	Histone methylation	H3K4me3 H3K36me3	Stadtfeld et al., 2012; Yu et al., 2018
	ND	Histone acetylation*	Histone lysine H3	Stadtfeld et al., 2012
		Histone acetylation*	H4K5	Huang et al., 2011
ALKB	FTO ALKBH5	RNA demethylation	N^6^-methyladenosine (m^6^A)	Tang et al., 2018
ND	ND	Expression of pluripotency and differentiation regulatory microRNAs	miR302/367 miR209/295 miR200 miR143	Gao et al., 2015; Yang et al., 2022

ND, not determined; * Indirect histone acetylation.

These dioxygenases are required for the differentiation of NSCs derived from embryonic midbrains into dopaminergic neurons, where vitamin C treatment increases 5-hydroxymethylcytosine (5hmC) content and decreases H3k27m3 in promoters of dopaminergic lineage differentiation genes, such as Nurr1 (He et al., 2015). In addition, the authors demonstrated that this mechanism is dependent on the expression and function of SVCT2, since the formation of dopaminergic neurons, as well as the changes in the content of 5hmC and H3k27m3, were decreased in embryos from SVCT2 knockout mice (He et al., 2015). The epigenetic action of vitamin C has also been evaluated during the gene reprogramming of human fibroblasts to induced pluripotent cells (IPSCs), where it has been shown that vitamin C substantially improves the efficiency and quality of reprogrammed cells compared to other antioxidants and that both processes depend on the action of Fe^2+^/α-KGDDs activated by vitamin C (Chung et al., 2010; Esteban et al., 2010; Wang et al., 2011; Table 1). Vitamin C also promotes DNA demethylation in the promoters of pluripotency genes such as Tbx3, Tcl1, and Esrrb and of the promoters of genes that encode miRNA-specific ESCs such as miR-290–295 and miR-17–92 clusters, as well as the DNA hydroxymethylation of the Dlk1–Dio3 region (Gao et al., 2014). In addition, vitamin C induces the expression of miRNA that regulates the Kdm6b, Klf13 and Sox6 genes, which inhibits cell differentiation and development. Thus, vitamin C plays an epigenetic role with a broad effect on the demethylation of the genome in promoters, maintaining the levels of all miRNA in the Dlk1–Dio3 region, as well as in pluripotency genes and ESC-specific miRNA (Gao et al., 2014).

**Signaling pathways activated by vitamin C.** Extracellular signal-regulated kinase 1/2 (ERK1/2) kinase phosphorylation has been demonstrated in N2a cells that overexpress SVCT2 and undergo morphological changes with the presence of numerous filopodia and processes (Salazar et al., 2016). In turn, the role of this kinase in the long phase of long-term potentiation (L-LTP) of synaptic plasticity is well established, where it maintains activation for several hours, increasing the expression of immediately early gene (Kandel, 2001; Sweatt, 2001; Thomas and Huganir, 2004). Thus, the uptake of AA by SVCT2, with the consequent phosphorylation and activation of ERK1/2, could be responsible for the expression of genes for neurogenesis, differentiation, and synaptic maturation observed in studies of DNA microarrays in stem cells, ventral precursors stimulated by AA (Lee et al., 2003; Shin et al., 2004; Yu et al., 2004) and in cortical neurons that overexpress SVCT2 (Salazar et al., 2021). However, it is likely that the activation of ERK1/2 by AA is an indirect effect dependent on a short and regulated imbalance of the intracellular redox state that would promote the presence of Ras-GTP and the consequent activation downstream of the protein kinase pathway activated by mitogens (MAPK) (Salazar et al., 2016). Recently, it has been proposed that the bidirectional functional role of vitamin C (promoting pluripotency or differentiation) depends on the action of SVCT2 as a novel receptor-like transporter (Han et al., 2021). To do this, together with mediating the intracellular uptake of AA, SVCT2 favors the autophosphorylation and activation of Janus kinase 2 (JAK2), which, in cells of the neural lineage, is physically associated with this transporter (Han et al., 2021). Once activated, JAK2 phosphorylates SVCT2 Tyr626, which serves as a site for the recruitment and activation of the transcription factor STAT2, thus increasing the expression of pluripotency genes (Han et al., 2021). In addition, JAK2 has an additional role that contributes to the cell reprogramming observed during the genesis of iPSCs treated with AA since it induces the global oxidation of 5mC and the consequent generation of 5hmC in DNA through the direct phosphorylation of TET3, thus increasing the global demethylation of the genome (Han et al., 2021). It was also proposed that JAK2 activation promotes the phosphorylation of an “x factor” that at the nuclear level induces the expression of neuronal differentiation genes (Han et al., 2021). This factor could be phosphorylated ERK1/2, activated by crosstalk, which is responsible for the increase in the expression of synaptogenesis genes previously observed in neuronal cells that overexpress SVCT2 (Salazar et al., 2016). Furthermore, the effects of JAK2 activated by vitamin C also regulate mitochondrial metabolism, where ROS generation is inhibited. This is achieved by direct activation of pyruvate dehydrogenase kinase 1 (PDHK1) by phosphorylation at the Tyr243 residue, which in turn phosphorylates the Ser232 residue of PDHK1 (E1 alpha subunits), thus inhibiting the PDH complex that catalyzes the oxidative decarboxylation of pyruvate (Han et al., 2021). In this way, AA and SVCT2 can activate signaling pathways in the cytosol and in particular organelles such as mitochondria and the nucleus to maintain or change cellular status.

## 7. Conclusion

Vitamin C can have multiple functions in the cells of the nervous system during development, mainly in stem cells. In radial glia, SVCT2 becomes apically polarized by incorporating AA from the CSF. Vitamin C stimulates neuronal differentiation by increasing neuritic growth in motor neurons. In adult neurogenic niches, SVCT2 was mainly detected in intermediate precursors, type C cells in the anterior lateral ventricle. In isolated neurospheres, SVCT2 maintains its expression, where it was concluded that vitamin C induced neural differentiation increased βIII-tubulin expression. During post-natal cerebellar development, SVCT2 is detected in granular precursor cells located in the external granular layer. C17.2 neural precursor cells (from cerebellum) supplemented with AA or SVCT2 overexpression for a period of 9 days induces a drastic decrease in nestin expression, increasing the neuronal marker βIII-tubulin, which accounts for the neuronal differentiation (Oyarce et al., 2018). Although there are no studies on the distribution of SVCT2 in SGZ of the hippocampal DG, it has been proposed that vitamin C enhances stem cells turnover, neuronal differentiation, and neuronal maturation. Overall, these studies demonstrate that vitamin C stimulates neuronal differentiation in all neurogenic niches studied. Consequently, it has been observed in guinea pigs that in scurvy condition, precursor cells proliferate less and fewer neurons are generated.

In the last decade it has been described that vitamin C has a potent epigenetic action by inducing the demethylation of DNA and histones, in the promoter region of genes involved in neurogenesis, differentiation and neurotransmission. However, it has also been described that, in a certain biological context, vitamin C maintains the pluripotency of mouse embryonic stem cells observing an increase in Nanog expression (Gao et al., 2013) dependent on JAK2/STAT2 phosphorylation. Vitamin C can also activate ERK1/2 kinase phosphorylation in neural cells, thus, the preferential activation of one signaling pathway or another will regulate pluripotency or differentiation of the neural cell. Defining how vitamin C stimulates or not differentiation mechanisms, acting in association with other molecules in development, for example retinoic acid, will allow a more precise understanding of the mechanism of action in these biological processes.

## Author contributions

KS, NJ, and FN conceived the ideas, concepts and wrote the manuscript. JS-G, VM, ER, IL, and LF contributed to the design of the schemes and wrote the manuscript. All authors have read and agreed to the published version of the manuscript.
